# Cross talk of tyrosine kinases with the DNA damage signaling pathways

**DOI:** 10.1093/nar/gkv1166

**Published:** 2015-11-05

**Authors:** Kiran Mahajan, Nupam P. Mahajan

**Affiliations:** 1 Tumor Biology Department, Moffitt Cancer Center, 12902 Magnolia Drive, Tampa, FL 33612, USA; 2 Drug Discovery Department, Moffitt Cancer Center, University of South Florida, 12902 Magnolia Drive, Tampa, FL 33612, USA; 3 Department of Oncological Sciences, University of South Florida, 12902 Magnolia Drive, Tampa, FL 33612, USA

## Abstract

Tyrosine kinases respond to extracellular and intracellular cues by activating specific cellular signaling cascades to regulate cell cycle, growth, proliferation, differentiation and survival. Likewise, DNA damage response proteins (DDR) activated by DNA lesions or chromatin alterations recruit the DNA repair and cell cycle checkpoint machinery to restore genome integrity and cellular homeostasis. Several new examples have been uncovered in recent studies which reveal novel epigenetic and non-epigenetic mechanisms by which tyrosine kinases interact with DDR proteins to dictate cell fate, i.e. survival or apoptosis, following DNA damage. These studies reveal the ability of tyrosine kinases to directly regulate the activity of DNA repair and cell cycle check point proteins by tyrosine phosphorylation. In addition, tyrosine kinases epigenetically regulate DNA damage signaling pathways by modifying the core histones as well as chromatin modifiers at critical tyrosine residues. Thus, deregulated tyrosine kinase driven epigenomic alterations have profound implications in cancer, aging and genetic disorders. Consequently, targeting oncogenic tyrosine kinase induced epigenetic alterations has gained significant traction in overcoming cancer cell resistance to various therapies. This review discusses mechanisms by which tyrosine kinases interact with DDR pathways to regulate processes critical for maintaining genome integrity as well as clinical strategies for targeted cancer therapies.

## INTRODUCTION

Tyrosine kinase (TK) signaling has garnered a lot of interest in recent years, principally in cancer research, due to the demonstrable success in developing precision drugs to target critical pathogenic drivers (1–4). Under regulated conditions, tyrosine phosphorylation acts as a rapid on-off switch in cells and is employed by the cellular signaling pathways to regulate growth, migration, adhesion, differentiation and survival. Conversely, constitutively active tyrosine kinase signaling cascades relay unrelenting growth and proliferation signals to promote tumor development, progression and metastasis in less than optimal environments. Tyrosine kinases are also known to be activated in cells upon DNA damage and in turn activate signal transduction networks required to restore cellular homeostasis (5–10). These networks comprise of proteins critical for DNA repair, cell cycle checkpoints, chromatin remodeling and restoration, miRNA processing, mRNA splicing and stability (Table 1). Understanding the mechanisms by which tyrosine kinases regulate DDR to impact cell fate in normal cells is essential to delineate their roles in cancer cell resistance to various DNA damaging agents.

**Table 1.** Tyrosine kinases–DDR interactions: phosphorylation sites and functional roles

Tyrosine kinase	DDR protein–phosphorylation site	DDR function	References
c-Abl	KAT5 at Y44	Binding to H3K9me3 residue and Activation of ATM by acetylation	(10)
c-Abl	Mdm2 at Y394	Disrupts the binding of MdmX to p53	(150,151)
	HDM2 at Y276	Regulates interaction with ARF	
c-Abl	MdmX at Y99	Disrupts the binding of MdmX to p53	(152)
c-Abl	p73 at Y99, p63 at Y149	Increase in apoptosis and cell death	(69,153,154)
c-Abl	MSH5	Interaction activates Abl kinase and tyrosine phosphorylation of MSH5	(155)
c-Abl	DNA PKCs (pYC-Terminal Domain)	Dissociates the DNA PKcs-Ku complex	(156)
c-Abl	BRCA1 (pYC-Terminal Domain)	BRCA1 negatively regulates Abl tyrosine kinase activity	(157)
c-Abl	Rad51 at Y315	Chromatin association during HRR	(108,109)
c-Abl	Rad52 at Y104	Ionizing radiation induced foci (IRIF) formation	(110)
c-Abl	YAP1 at the Y357	Regulates p73 dependent apoptosis	(80)
c-Abl	ATM and DNA PKcs	IR induced response	(65,100,104)
c-Abl	HIPK2 at Y360	Regulates p53 dependent apoptosis	(6)
ACK1/TNK2	AR at Y267	Upregulates AR mediated ATM expression	(56,72)
ACK1/TNK2	KDM3A at Y1114	Regulates HOXA1 gene expression	(142)
EGFR	H4 at Y72	Chromatin modulation	(57)
EGFR	ATM at Y370	DNA synthesis and repair, IR induced foci formation	(158)
EGFR	DNAPKcs	NHEJ/radioresistance	(39)
EGFR	PCNA at Y211	Mismatch repair	(61)
IGF-1R	IRS-1	Disrupts IRS-1-Rad51 complex. Required for nuclear translocation of Rad51 during HRR	(30)
Lyn	CDK1, DNA-PK and PKCδ	IR induced activation and association of Lyn - with CDK1. Lyn activates DNA-PK and PKCδ	(63,75–78)
Rad53	H3 at Y99	Regulate histone levels	(87,88)
Src, Fyn, and c-Abl	AKAP8	Dissociation from chromatin and nuclear matrix	(121)
Src	ATR/Chk1	Termination of DNA damage response	(31)
WSTF	H2A.X at Y142	Radiosensitization	(112)
WEE1	CDK1 at Y15	G2/M arrest	(159,160)
WEE1	H2B at Y37	Global histone synthesis	(92)

The tyrosine residues in DDR proteins specifically modified by the individual tyrosine kinases are shown in the table. The functional outcome of the tyrosine phosphorylation on the activity of the DDR protein or the corresponding DNA repair pathway is detailed above. Little cross substrate phosphorylation is observed between individual tyrosine kinases. Each DDR protein appears to be uniquely targeted by a specific tyrosine kinase at distinct tyrosine residues.

**Figure 1.**
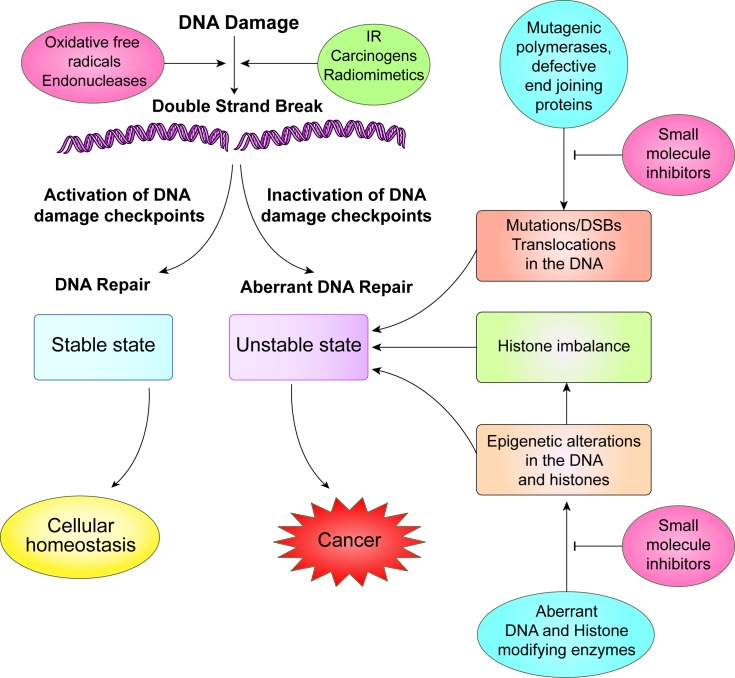
Integrity to the genome is under threat by a variety of exogenous and endogenous agents that activate DNA damage checkpoints. Chromatin alterations can also activate DNA damage signaling pathways. Activated checkpoint kinases, ATM or ATR arrest the cells at a specific stage in the cell cycle and allow time for repair. DNA double strand breaks caused by ionizing radiation may be repaired either by the homologous recombinational repair pathway (HRR) or the non-homologous end joining pathway (NHEJ). In addition, eukaryotic cells face a variety of other situations which could also lead to an unstable genomic state, e.g. mutagenic DNA polymerases, aberrant activity of the end joining proteins and mutations in the DNA and histone modifying enzymes. Small molecule inhibitors targeting these aberrant proteins have emerged to be a therapeutic option which could not only restore genome stability but also inhibit tumor growth by radiosensitization.

DNA double-strand breaks (DSBs) pose a grim threat to cells as loss or gain of genetic material, mutations and chromosome rearrangements due to improper repair can be a primer for malignant transformation (11–14) (Figure 1). In normal cells, DSBs are repaired with high fidelity by members of the homologous recombination (HR) pathway which restore the genetic integrity using a donor template (15). In contrast, when the DSBs are acted upon by members of the non-homologous end joining (NHEJ) pathway, the ends are joined directly with little or no homology and the process is thus error prone. The classical NHEJ or the canonical NHEJ (c-NHEJ) does not rely on the availability of homologous ends for joining DSBs and is catalyzed by Ku complex in conjunction with seven other core members of NHEJ (16–18). Albeit to a lesser degree, some homology-independent repair occurs in the absence of these Ku proteins, and is referred to as the microhomology-mediated end joining (MMEJ), or alternative NHEJ (alt-NHEJ) (19). Even though DNA damage is the initiator of the HR, cNHEJ, and Alt-NHEJ pathways, the activity of the DDR proteins operating in these pathways may be further regulated by post-translational modifications by tyrosine kinases (Table 1). Conversely, inhibition of tyrosine kinase signaling significantly enhances the induction of radiation-induced apoptosis, prevents radiation-induced invasiveness and reverses the radioresistance of tumor cells by interfering with DNA repair (20,21).

**Figure 2.**
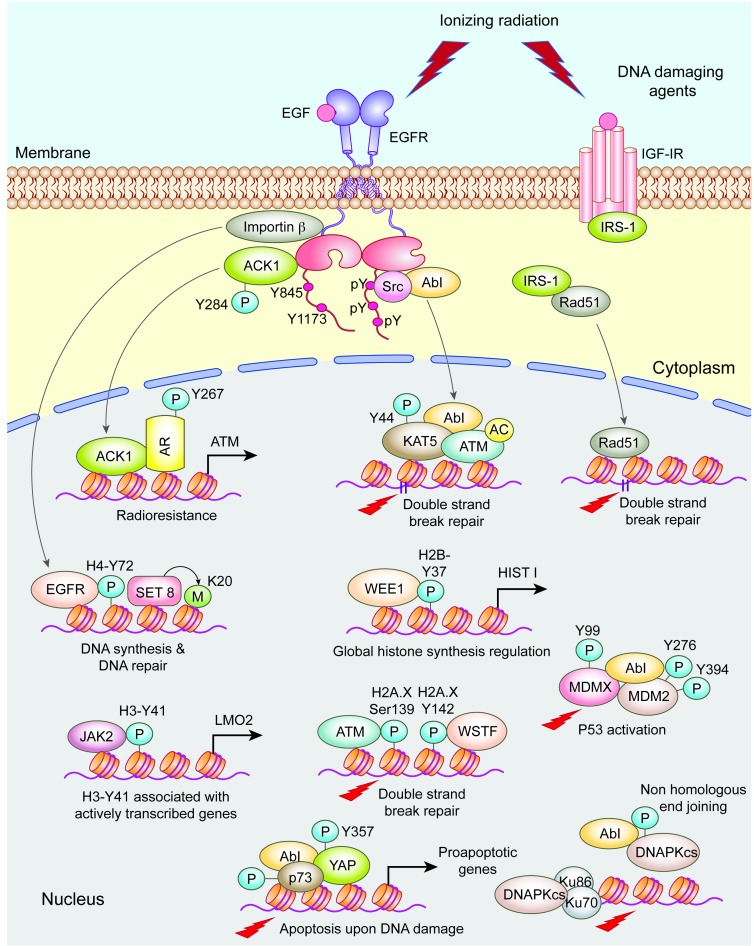
Activation of the DDR proteins in cancerous cells is intricately linked to activated tyrosine kinases and their downstream signaling partners, some of which are also tyrosine kinases. Transmembrane tyrosine kinases, such as the EGFR and IGF-1R are activated by ionizing radiation or by growth factors. Activated EGFR undergoes auto-phosphorylation that in turn recruits and activates non-receptor tyrosine kinases (NRTKs), Src and ACK1/TNK2. These NRTKs shuttle between the cytosol and the nucleus, relaying signals from multiple transmembrane RTKs directly to the nuclear compartment to activate specific DDR pathways. A nuclear form of EGFR modifies, the histone H4 at Tyr72 which functions as a docking site for the chromatin modifying enzymes SET8 and SUV4–20H that methylate H4 lysine 20. In contrast to EGFR, in IR-treated cells, nuclear non-receptor tyrosine kinase Abl directly phosphorylates the histone lysine acetyl transferase KAT5/TIP60 by modifying the Tyrosine 44 in the chromodomain. KAT5 acetylates and activates ATM at DSB sites and loss of KAT5 Tyr44-phosphorylation is associated with increased sensitivity to ionizing radiation. ATM phosphorylates H2A.X at Ser139 residue. In addition, Abl exhibits another response mode- it antagonizes YAP1 oncogenic function by phosphorylating it at Tyr357, which works in conjunction with the tumor suppressor p73 to promote apoptosis in irradiated cells. Abl also antagonizes the negative regulators of the tumor suppressor p53, MDM2 and MDMX, in response to DNA damage. Abl modification of the catalytic subunit, DNAPKCS interferes with the formation of active DNAPK-Ku complex. A non-receptor tyrosine kinase JAK2 utilizes its epigenetic activity wherein it modifies Histone H3 at Tyr41 excluding the binding of heterochromatin protein HP1 which resulted in elevated expression of oncogene *lmo2*. WEE1directly phosphorylates the histone H2B at Tyr37, consequently terminating global histone transcription at the end of S phase to maintain histone homeostasis. ACK1 is another NRTK that not only phosphorylates Androgen receptor (AR), but also was recruited to a distinct set of cell cycle and DNA damage checkpoint genes, including ATM to confer radioresistance in prostate cancer cells. IGF-IR regulates HRR by modulating the interaction of the insulin receptor substrate 1 (IRS-1) with Rad51 following radiation-induced DNA damage.

Oncogene induced replication stress impairs replication fork progression and therefore activates the DNA damage checkpoints, which function as an anti-cancer barrier (8,22,23). These checkpoints are critical to stall cell cycle progression to allow time for repair, so that cells do not linger with damaged DNA that potentiates genome instability phenotypes (11,24). Cells may either enter replicative senescence or undergo mitotic arrest in the presence of broken chromosomes and shortened telomeres (25–27). However, cancer cells circumvent this anti-tumor barrier by overexpressing positive regulators or mutating the negative regulators of tumorigenesis (28). Tyrosine kinases are also activated in cells following exposure to radiation, interact with DNA repair and checkpoint pathways, to promote survival or apoptosis (29,30). In addition, tyrosine kinases may directly terminate activated checkpoints (31,32). Further, both receptor and non-receptor tyrosine kinases epigenetically regulate DNA damage signaling by modifying the core histones as well as chromatin modifiers (Figure 2). Thus, the cross talk of tyrosine kinases with the DNA Damage Response (DDR) proteins presents a conundrum of oncogene and tumor suppressor interactions and consequently adds complexity into a precisely orchestrated control of genome stability (24). Consequently, deregulated tyrosine kinase driven genomic and epigenomic alterations have profound implications in cancer, aging and genetic disorders. This review discusses novel mechanisms by which tyrosine kinases regulate DDR pathways and the physiological and clinical implications of targeting these pathways in cancer.

### Receptor tyrosine kinases are activated by DNA damage

Approximately, 20 receptor tyrosine kinase (RTK) subfamilies and at least ten distinct groups of non-receptor tyrosine kinases (NRTK) have been identified in humans, which correspond to about 100 kinases (33). Transmembrane tyrosine kinases, such as the Epidermal Growth Factor Receptor (EGFR) or Insulin like Growth Factor 1 Receptor (IGF-1R) are activated not only in response to extracellular signals originating from the binding of respective ligands or growth factors, but also upon exposure of cells to ionizing radiation, independent of ligand stimulation (Figure 2) (30,34–37). For example, the phosphorylation of EGFR at residues Y845 and Y1173 was found to be strongly induced by ionizing radiation (IR) (38). IR activated EGFR is transported in a Src kinase dependent manner, and interacts with the NHEJ protein, DNAPK (38). EGFR may be also internalized in the nuclear compartment as a component of the lipid rafts or due to its interaction with importin beta (39) (Figure 2). Blocking the nuclear transport of EGFR by Erbitux, markedly impaired radiation associated activation of DNA-PK and increased cellular radio-sensitivity demonstrating a direct regulation of NHEJ repair by RTKs (38,40). Moreover, unlike the selectivity seen with the binding of specific growth factors, ionizing radiation randomly activates, albeit to a lesser degree, all members of the EGFR family, ErbB receptors 1–4 (35–37). The random nature of oxidative damage induced by ionization has been suggested as a mechanism for this indiscriminate EGFR family activation (37). This response occurs in a biphasic manner as secondary activation of the EGFR is observed followed initial radiation-induced activation in cancer cells and may be mediated by radiation-induced cleavage and autocrine action of TGFα (41). A proposed mechanism by which EGFR promotes survival in response to radiation in some genetic backgrounds such as K-Ras mutated cell line is the activation of PI3K/AKT signaling pathway that transduces signal to DNAPK stimulating DSB repair (29). Further, treatment of lung cancer cells with EGFR specific small molecule inhibitor gefitinib (Iressa), strongly inhibited the DSB repair after ionizing radiation, indicating that EGFR promotes radiation response by augmenting DNA repair capacity of cancer cells (42).

Ionizing radiation also activates IGF-1R and depletion of IGF-1R or pharmacological blockade of this pathway increased cellular radiosensitivity by modulating DSB repair (30,34) (Figure 2). Additionally, in human neuroblastoma cells, expression of XRCC4, a central component of NHEJ, was significantly upregulated upon overexpression of TrkA receptor tyrosine kinase leading to increased NHEJ activity (43). Consistently, TrKA expression correlated with faster repair of IR-induced DSBs, clinically correlated with increased chromosome stability and favorable prognosis of childhood neuroblastomas. Combined, these studies suggest that activation of tyrosine kinase receptors in response to DNA damage contributes to the genomic stability of cancer cells.

EGFRvIII, a deletion mutant of EGFR that lacks the extracellular, ligand-binding domain, is constitutively active and exhibits higher level of autophosphorylation in response to IR (44). In contrast to activated EGFR which stimulates both the RAS/RAF/MAPK and PI3K/AKT pathways, EGFRvIII appears to preferentially and robustly activate only the PI3K/AKT signaling cascade (45–47). Consistently, inhibition of PI3K/AKT signaling impaired DSB repair and enhanced radio sensitivity of brain (GBM) as well as breast cancers (48–51). Interestingly, in the light of the recent reports that demonstrate radiation induced AKT translocation to the nucleus and association with DNAPK at DSBs, it is likely that hyperactivation of DNAPK by EGFRvIII/AKT signaling could potentiate DNA repair in cancer cells, opening new therapeutic options to overcome radiation resistance in cancer cells (52–54). In addition, tyrosine 176 phosphorylated AKT is known to translocate to the nucleus in a PI3K-independent and ACK1 tyrosine kinase dependent manner (55). While activated ACK1 expression is correlated with radioresistance in prostate cancer cells (56), the role of ACK1-pY176AKT signaling in radioresistance is not yet explored.

The internalization of TKs to cues of radiation damage suggests that cells may be using this mechanism to prevent additional growth factor induced signaling cascades until the DNA damage is repaired. Some of the studies described below provide further mechanistic insights into how activated oncogenic tyrosine kinases fine tune the repair of radiation induced DNA damage.

### Receptor tyrosine kinases epigenetically regulate DDR

Recent studies have uncovered novel epigenetic underpinnings of the RTK-DDR cross talk. The nuclear form of EGFR has been reported to directly modify the core histone H4 at tyrosine 72 (57). The H4 Y72-phosphorylation functions as a docking site for another group of chromatin modifying enzymes SET8 and SUV4-20H that subsequently methylate lysine 20 (Figure 2). Consequently, the H4Y72/H4K20 dual motif directs transcriptional programs for optimal DNA synthesis and DNA repair in cells, implicating RTKs in DNA metabolic activities. Conversely, the H4 Y72 peptide disrupted EGFR-histone H4 interaction and was able to suppress breast cancer xenograft tumor growth (57). With the discovery that the IR-induced checkpoint proteins, mammalian 53BP1 (and its ortholog Crb2 in *S. pombe*), interact with methylated H4K20 at DSB sites via their tudor domains, the loss of H4 Y72-phosphorylation in delaying cell cycle progression could be due to persistent and unrepaired DSBs that eventually lead to apoptosis (58,59). In addition, EGFR is reported to stabilize the binding of the Proliferating Cell Nuclear Antigen (PCNA) to the chromatin by modifying it at Y211 (60). This phosphorylation of PCNA promotes misincorporation of nucleotides during DNA synthesis by inhibiting the activity of mismatch repair proteins at the initiation step (61). Accordingly, interference with the activity of pY211-PCNA led to S-phase arrest, inhibition of DNA synthesis, cytotoxicity and decreased tumor growth of prostate cancer xenograft tumors in mice (62). Collectively these studies emphasize the notion of a direct control of regulation of the DNA damage signaling cascade by RTKs during malignant transformation.

### Non-receptor tyrosine kinases regulate DNA damage checkpoint pathways

DDR proteins are also targets of non-receptor tyrosine kinases (NRTKS), such as Abl-1, ACK1 (TNK2) and Src family of tyrosine kinases (Table 2 and Figure 2). Importantly, NRTKs shuttle between the cytosol and the nucleus, and have emerged to be a major intermediary in cellular signaling capable of relaying signals from activated RTKs directly to the nuclear compartment, to activate specific DDR pathways (31,56,63,64).

**Table 2.** Tyrosine kinases in the DDR network: Alteration frequencies in various cancers

Tyrosine kinase	Alteration frequency	Frequently mutated cancers	Amplification/deletion	Inhibitors/drugs
ABL1	4% SAC, 2% AML	Stomach adenocarcinoma (SAC), Acute myeloid leukemias (AML)	Amplifications and missense mutations, Gene fusion	Imatinib
ACK1 or TNK2	26% LSCC, 27% OSC, 19% HNSCC, 9% SAC, 8% DLBCL	Lung squamous cell carcinoma (LSCC), Ovarian Serous Cystadenocarcinoma (OSC), head and neck squamous cell carcinoma (HNSCC), stomach adenocarcinoma(SAC), diffuse large B cell lymphomas (DLBCL)	Amplifications	AIM-100, (*R*)-9b
EGFR	53% GBM, 8% LSCC, 14%, HNSCC, 11% SAC	Glioblastoma (GBM), lung squamous cell carcinoma (LSCC), head and neck squamous cell carcinoma (HNSCC), stomach adenocarcinoma (SAC)	Amplifications and missense mutations	Gefitinib, Lapatinib, Iressa, Cetuximab/Erbitux, Nimotuzumab
IGF1R	10% SAC, 9% OSC, 7%, LHC, 7% CAC	Stomach adenocarcinoma (SAC), ovarian serous cystadenocarcinoma (OSC), liver hepatocellular carcinoma, colorectal adenocarcinomas (CAC)	Amplifications and missense mutations	Cixutumumab (IMC-A12), Dalotuzumab (MK-0646; h7C10), Linsitinib (OSI-906)
EPHA5	12% LAC, 10% SCLC, 9% SAC,	Lung adenocarcinoma (LAC), small cell lung cancer (SCLC), stomach adenocarcinoma (SAC),	Mutations, amplifications	none
FYN	15% DLBCL	Diffuse large B cell lymphomas (DLBCL)	Deletions	Various Src inhibitors
WEE1	<1%	Glioblastomas, sarcomas	Deletions and missense mutations	AZD1775

The tyrosine kinases that participate in DNA damage response signaling are reported to be frequently mutated in a number of malignancies. The frequency and type of known genetic alteration of the tyrosine kinase, the cancer subtype where it is most frequently mutated and the small molecule inhibitors/drugs targeting each tyrosine kinase signaling pathways are shown.

Reference for mutation frequencies: www.cBioPortal.org.

Abl-1 is a versatile kinase that is activated in cells exposed to genotoxic stress and directly phosphorylates a number of key cellular proteins to promote DNA repair, cell cycle arrest or apoptosis (Table 1 and Figure 2) (65,66). Abl-1 modulates the activity of a number of substrates; Rad51, Rad52, Msh2, ATM, DNAPKCs, BRCA1 and MDMX to regulate some of the major pathways of DNA DSB repair (67,68) (Table 2). Abl-1 also functions as a tumor suppressor in response to radiation induced DNA damage by phosphorylating p73 at Y99 and transactivating and promoting p73 dependent apoptosis (69,70). Recently, it was uncovered that in addition to p73, Abl-1 can also phosphorylate HIPK2 at Tyr360 facilitating p53 dependent apoptosis upon DNA damage (Table 1) (6). Significantly, the DNA damage-induced apoptosis and the activation of the tyrosine kinase c-Abl were regulated by the tumor suppressive Hippo Lats2 kinase depending on the cell density (9). Other mechanisms by which Abl-1 regulates DNA damage checkpoints and DDR proteins to promote genome integrity have been extensively covered (66) and will not be discussed in depth in this article.

In contrast to Abl-1, NRTKs such as ACK1 and the Src family of tyrosine kinases have been shown to regulate radioresistance by indirect mechanisms. ACK1/TNK2 tyrosine phosphorylates AR at Y267 in androgen deprived conditions of prostate cancer cells (56,71,72). Mechanistically, ACK1/pY267-AR complex is recruited to the upstream elements of a distinct set of cell cycle and DNA damage checkpoint genes in an androgen-independent manner (Table 1) (56,72). Notable among these is an upstream binding site in the *ATM* (Ataxia Telangiectasia Mutated) gene, DNA damage checkpoint regulator, that contains ARE-like (androgen responsive elements) sites (56,73). Consequently, activated ACK1 promoted radioresistance of prostate cancer cells and conversely, a small molecule ACK1 inhibitor, e.g. AIM-100 blocks ATM dependent DNA damage induced G2/M arrest, resulting in the accumulation of cytotoxic DSBs (Table 2).

The Src family of NRTKs including Src, Fyn and Lyn may also influence the DDR responses (22,31). Src is known to be phosphorylated upon IR treatment (74). Moreover, the radiation dependent activation of EGFR was found to be Src dependent in some tumor cell lines (38). Intriguingly, the mechanism of action of Src family of kinases appears to be distinct from ACK1, as these kinases oppose the activity of the checkpoint kinases. Just as activation of the DDR pathways is tightly regulated, its deactivation also appears to be precisely controlled within the cells (22). Accordingly, the recovery from G2/M checkpoint arrest via dephosphorylation and degradation of the checkpoint kinases following completion of DNA repair is suggested to be dependent on the activity of the Src family of tyrosine kinases (31). Although, the exact mechanism is not clear, one putative scenario that has been proposed is the silencing of ATR/Chk1 signaling cascade through an increase in the inhibitory nuclear tyrosine phosphorylation events. These findings may illuminate its well established role as an oncogene wherein it has been demonstrated to have a role in cell proliferation, invasion and motility. In activated Src expressing cancer cells, the cells rapidly recover from stalled replication forks, such as those caused by oncogene-induced replicative stress and resume cell cycle progression (31). Consistent with these observations, Src inhibitors induce a prolonged G2/M arrest and growth inhibition or apoptosis (31). Additionally, the Lyn tyrosine kinase, a member of Src family is activated by ionizing radiation and mitomycin C treatment (75,76) and interacts with the cell division cycle protein Cdc2, DNAPK and protein kinase C delta (PKC delta) in irradiated cells (63,77,78). Thus, the regulation of DDR pathways by oncogenic cytosolic kinases suggests that cancer cells effectively use these survival kinases to circumvent radiation induced apoptosis.

### Epigenetic interactions of non-receptor tyrosine kinases with DDR proteins

Cells activate the DDR pathways following exogenous and endogenous DNA damage. However, it is not only a physical damage to the DNA but the chromatin alterations can also trigger DDR pathways. These chromatin alterations can manifest either as structural alterations due to changes in nucleosome packaging, histone imbalance, or due to the removal or addition of specific epigenetic modifications on histones. DNA DSBs induced by the topoisomerase II inhibitor, Adriamycin, can also promote translocation of c-Abl into the nucleus, causing specific hypoacetylation of H4K16 residue in a tyrosine phosphorylation dependent manner (79). Interestingly, hyperacteylation of histones by trichostatin A, a histone deacetylase inhibitor, also activates Abl dependent ATM activation, suggesting that Abl is sensing different epigenetic alterations (10). Further, the bi-directional Abl/ATM cross talk may be a context dependent signaling event that underlies chromatin accessibility at loci with increased H3K9 trimethylation, and is the basis for differences in DDR responses at euchromatin versus heterochromatin loci (10). Notably, increased hyperacetylation and loss of HP1 binding has been shown to augment the Tyr-phosphorylation of histone acetyl transferase, KAT5 (10). The binding of KAT5 to H3K9me3 leads to acetylation and activation of ATM that is needed for Chk2 and p53 phosphorylations to promote DNA damage induced cycle arrest (Table 1). In addition to activating the p53 tumor suppressor dependent apoptotic pathway, c-Abl also promotes p73-YAP dependent apoptosis by negating the oncogenic activity of YAP in response to DNA damage by phosphorylating it at Tyr357 and preventing the activation of the TEAD family of transcription factors (Table 1 and Figure 2) (80). These studies highlight the ‘decision maker’ function of c-Abl to determine whether to activate apoptotic program or initiate the DNA repair process.

### Epigenetic interactions of non-receptor tyrosine kinases with histones to maintain genome integrity

Chromatin duplication is a singularly vulnerable phase in cell cycle. Any incompletely replicated DNA or excess histones can perturb histone/DNA stoichiometry, disrupt chromosome homeostasis to promote genome instability and mitotic catastrophe. Additionally, histone imbalance can alter stable gene expression profiles in cells by inducing epigenetic alterations that are often linked to cancer and developmental disorders (81–86). There are at least two examples by which tyrosine kinase and proteins with tyrosine kinase like activity maintain histone/DNA stoichiometry of the chromatin. The first example is the DNA damage checkpoint kinase from the budding yeast, *Saccharomyces cerevisiae*, Rad53, (homolog of CHK2 in humans) which regulates histone homeostasis within cells (87). Rad53 harbors both serine/threonine and tyrosine kinase like-activity and protects cells from cytotoxic effect of excess histones. Mechanistically, it phosphorylates excess non-chromatin bound histone H3 at Tyr99 and flags it for proteosomal degradation (88,89). Importantly, its ability to prevent excess non-nucleosomal histones from interfering with the initiation of DNA replication was found to be independent of its role as an effector of the Mec1 mediated replication and DNA damage checkpoint activity in stabilizing stalled replication forks (90). Consistently, deletion of a major copy of the H3-H4 genes in *S. cerevisiae* suppressed the synthetic lethality associated with replication defective mutant of Rad53 (*rad53-rep*) and the cell division cycle protein 7 *(cdc7-1)* (homolog of CDC7Hs in humans). Together, these studies provide insights into how tyrosine and threonine kinase activity of Rad53 may be employed to regulate two distinct protein–protein interaction networks within cells (90,91).

The second example is WEE1 kinase, which directly phosphorylates the histone H2B at Tyr37, to terminate global histone transcription at the end of S phase (92). Mechanistically, the histone transcriptional repressor, HIRA acts as the ‘reader’ of the pY37-H2B epigenetic marks and its recruitment excluded the histone transcriptional co-activator, NPAT, to suppress global histone mRNA synthesis. Importantly, the WEE1-specific small molecule inhibitor, MK-1775 (also known as AZD1775), reversed histone transcriptional suppression by blocking this WEE1 epigenetic function (Table 1 and Figure 2). The role of H2B Tyr37-phosphorylation in G2/M arrest has not been elucidated so far. Future studies investigating its requirement in IR-induced or replication stalling induced WEE1 checkpoint functions may reveal whether anti-tumor activity of WEE1 inhibitors is a combination of suppression of WEE1 epigenetic activity and blockade of cell cycle progression.

JAK2, another NRTK, phosphorylates histone H3 at tyrosine 41 to regulate transcription by marking several active promoters, including the leukeomogenic oncogene Imo2, which regulates hematopoietic stem cell development in normal cells (93). Increased levels of phosphorylated H3Y41 as well as JAK2 activity is seen with GBMs and treatment with JAK2 inhibitors was found to overcome radioresistance of GBMs treated with temozolomide and IR (94). Overall, the ability of NRTKs to interact directly with the chromatin in normal and cancer cells has opened doors to further interrogate their direct roles in regulating DNA damage signaling and chromatin integrity in response to radio- and chemotherapy.

### Tyrosine kinases in DSB repair

Tyrosine kinases also influence DNA DSB repair by directly modifying proteins involved in HR or NHEJ following DNA damage. EGFR/AKT/ERK signaling is activated and protects tumor cells from cytotoxic doses of ionizing radiation induced DNA damage (35,37,41). Further, somatic mutations in EGFR tyrosine kinase have been shown to differentially modulate DNA double strand break repair as seen by differences in the kinetics of resolution of IR induced H2AX foci and enhanced clonogenic survival compared to the wild type EGFR (35,37,41). In addition to EGFR, IGF-1R also regulates DSB repair mediated by both, non-homologous end-joining and homologous recombinational repair proteins (HR) (30,34,95–97). Mechanistically, IGF-IR tyrosine phosphorylates a major downstream substrate, the insulin receptor substrate 1 (IRS-1) upon IGF-1 stimulation (30). IRS-1 interaction with Rad51 is negatively regulated by tyrosine phosphorylation in the cytosol. IGF-1 stimulation disrupts the IRS-1-Rad51 complex promoting Rad51 entry into the nucleus (Figure 2). Conversely, attenuation of IGF-1R signaling lead to diminished translocation of Rad51 to the sites of damaged DNA, compromising homologous recombination-directed DNA repair (Table 2) (30). In contrast in lung cancer cells, IGF-1R inhibition decreased the radiation-induced Ku-DNA-binding in a p38 MAPK dependent manner supporting a role for IGF-1R signaling in NHEJ mediated DSB repair (34).

Although the exact mechanism is not known, expression of TrkA tyrosine kinases accelerates IR induced DNA DSB repair by upregulating the expression of the NHEJ protein XRCC4 in neuroblastoma cell lines (43). Similarly, fusion tyrosine kinases drive drug resistance in hematopoietic cells by utilizing multiple routes *i.e*. STAT5 dependent upregulation of RAD51, recruiting it to repair DSBs by homologous recombination, promoting anti-apoptotic activity of BCL-XL and delaying cell cycle progression (98).

c-Abl tyrosine kinase regulates DNA repair by multiple mechanisms (Table 1). The instantaneous activation of c-Abl kinase upon IR-induced damage is primarily mediated by ATM via phosphorylation at Ser465 or may be mediated by DNAPK at an unknown site (65,99,100). Intriguingly, this DDR-TK regulation appears to be bidirectional, as ATM activation is itself regulated indirectly by c-Abl activity when complexed with KAT5/TIP60 (10). In IR-treated cells, Abl phosphorylates the histone lysine acetyl transferase KAT5/TIP60 by modifying the Tyr44 in the chromodomain (Table 1). Loss of KAT5 Tyr44-phosphorylation is associated with increased sensitivity to ionizing radiation. ATM and KAT5 form a distinct complex (101) that is recruited near DSB sites, displacing the heterochromatin protein 1 (HP1) at H3K9me3 in a MRN (Mre11-Rad50-Nbs1) dependent manner (102). Abl is acetylated by TIP60/KAT5 in response to DSBs at K921 which is required for IR induced apoptosis (103). c-Abl has also been shown to directly interact with and phosphorylate DNAPK, causing the dissociation of DNAPK/Ku protein complex from the DNA and attenuating NHEJ repair (104). Interestingly, in cells exposed to IR, c-Abl/BRCA1 interaction is also compromised (105). Further, c-Abl activated by IR was also shown to mediate phosphorylation of PI3K and mTOR, causing the inhibition their kinase activity (106,107). IR activated c-Abl tyrosine phosphorylates Rad51 at Tyr315 which increases association with Rad52 and chromatin in the recombination complex, in an ATM dependent manner (108,109). Interestingly, Rad52 is also phosphorylated at Tyr104 and this modification is required for the formation of ionizing radiation induced foci (IRIF) (110,111). These results suggest that Abl-1 is a critical regulator of DNA damage responses by post-translationally modifying epigenetic and non-epigenetic regulators to promote DNA repair or apoptosis.

An off-on tyrosine/serine phosphorylation switch in the tail of variant histone, Histone H2A.X, directs repair in response to DSBs (112,113). ATM phosphorylates the histone variant, H2A.X, at Serine 139 (γH2A.X) in response to DNA DSBs. γH2A.X functions as an active epigenetic mark for the recruitment of the mediator/adaptor proteins and assembly of multimeric protein complexes critical for DNA damage signaling and repair (114). Interestingly, H2AX is modified constitutively at Tyr142 by a non-canonical tyrosine kinase, WSTF (William-Beuren Syndrome Transcription Factor) (Table 1 and Figure 2) (112). These pY142 H2A.X marks are progressively erased following DNA damage and are suggested to be important to direct cells to choose between DNA repair or apoptosis (113–115). Intriguingly, mutation in the Y142 sensitizes chicken DT-40 cells to IR-induced DNA damage, suggesting that in wild-type cells, Tyr142-phosphorylation co-ordinates with increased Ser139-phosphorylation to facilitate DNA repair, before its complete removal on the chromatin. MCPH1, a tandem BRCT domain containing protein was found to specifically interact with the diphosphorylated-H2A.X (113–115). Paradoxically, a complete loss of both Ser139 and Tyr142 phosphorylations at the C-terminal tail of H2A.X appears to alleviate the radiation sensitivity of the Y142 mutant alone. The observation that another tandem BRCT domain protein, 53BP1, previously shown to colocalize with γH2A.X at damage sites, persisted at IRIFs in the H2AX-Ser139/Tyr142 double mutants, suggested that assembly at DNA ends may be compensated by BRCT domain containing proteins that are recruited through other histone modifications at the DSBs. In one example, MMSET a histone methyltransferase was found to interact with the BRCT domain of MDC1, an adaptor protein that binds to phosphoS139-H2A.X. Subsequently, MMSET methylates H4K20 locally at DSB sites and this methylation is critical to recruit 53BP1 (59). However, MDC1 recruitment is dependent on H2AX S139 phosphorylation. Therefore, identification of other histone modifications or chromatin regulators that can recruit MDC1 in the absence of H2AX phosphorylation would be the key to identify alternative mode of assembly of checkpoint proteins and DNA repair factors at DSB sites. Indeed, a recent report has identified a role of H2A.X methylation at lysine 134 in regulating γH2A.X levels in cancer cells, loss of which resulted in severe sensitivity to IR, and DSB inducing chemotherapeutic agents, Cisplatin and Doxorubicin (116).

A chromatin interacting protein, localized to DNA lesions is the hPso4/Prp19, which was originally identified for its ability to interact with the BRCT domain of the template independent DNA polymerase, terminal deoxynucleotidyl transferase (TdT), but has subsequently been shown to be phosphorylated by ATM following DNA damage (117,118). Importantly, it is upregulated in response to high doses of radiation and its interaction with chromatin is enhanced in the presence of DNA damage. hPso4 localizes with Nbs1 and Metnase at DSBs, and promotes DNA end-joining following IR induced DNA damage (117,119). Conversely, cells with loss of hPso4 protein display sensitivity to DSB inducing agents (117,120). Whether hPso4 interacts with the BRCT domain of MDC1 at DSB sites to promote DNA repair remains to be seen.

A role for the Src family of kinases, Src, Fyn, and Abl kinases, in causing global nuclear structural changes by altering chromatin has been uncovered by many groups. These kinases regulate the interaction of Protein kinase A anchoring protein 8 (AKAP8, also known as AKAP95) with the chromatin and the nuclear matrix by modifying it by tyrosine phosphorylation (121). AKAP8/AKAP95 is a zinc finger containing protein with RNA binding activity (122) that interacts with the RII-alpha regulatory subunit of protein kinase A (PKA) and implicated in chromosome condensation during mitosis (123). Interestingly, similar to ACK1, Src is also known to interact and modify AR to modulate expression of the AR target genes in prostate cancer cell lines (124). Whether tyrosine phosphorylated Src can directly regulate DDR is not known, nor is it clear whether it would modulate KDM3A or other related chromatin modifiers, particularly under conditions where ACK1 is inhibited. However, its ability to shuttle between the cytosol and nucleus is suggestive of this possibility. Collectively, these studies collectively paint a picture of the involvement of NRTKs in direct interactions with DDR in adverse treatment-rich environment to promote hormonally regulated cancers.

### Physiological and clinical relevance of DDR and TK crosstalk

The physiological significance of tyrosine kinase interaction with DDR pathways is still fairly underexplored. Mice with genetic deficiency in nuclear TK, WEE1 or in the DNA repair proteins, Rad51 and PSO4/PRP19 are embryonic lethal, suggesting that some of these interactions are essential for early development (125–128). Despite the inherent genome instability within cancer cells, oncogenic tyrosine kinases employ the DNA damage response (DDR) pathways to bypass checkpoints in conjunction with activating other survival pathways promoting tumor growth. Cross-cancer alteration analysis for Abl-1, EGFR, Fyn, LCK, Src, ACK1 (TNK2) tyrosine kinases reveal that these genes are frequently altered in cancers, e.g. EGFR and TNK2 are recurrently amplified and mutually exclusive in a majority of the cancer cases (Table 2). This table not only details the alteration in those tyrosine kinases that are involved in DDR, but also shows that many of the tyrosine kinase inhibitors or TKIs have been employed in clinical setting as cancer drugs. Not surprisingly, TKIs have emerged as one of the most common therapeutic strategy for the treatment of a variety of cancers, either alone (1–4) or in combination with radiotherapy (23). Consistently, EGFR activation is associated with tumor radioresistance and poor prognosis in GBMs and lung cancers (35,37,41). Conversely, blockade of EGFR/AKT/ERK signaling with small molecule inhibitors compromised DSB repair by blocking NHEJ and HRR. Certain cancers associated with activating mutations of EGFR also appear to influence phosphorylation and expression of members of DDR response pathways, including phospho-DNAPK, ATM, and RAD51 foci, suggesting that both HRR and NHEJ pathways may have roles in conferring radioresistance.

Frequently considered are the TKIs, gefitinib, lapatinib and erlotinib to block EGFR, as activation of this tyrosine kinase was found to correlate with radioresistance and survival. Gefitinib and lapatinib have been reported to inhibit DNA DSB repair in HER2 amplified breast cancer cell lines (129). High expression of RTKs, e.g. EGFR expression could lead to a higher degree of radiosensitization with erlotinib when combined with radiation (130). Further, EGFR inhibitors or blockade of the EGFR protein with selective monoclonal antibodies sensitized cancer cells to radiation therapy by blocking the repair of damaged DNA (39) and improved clinical outcomes in a subset of patients with head and neck squamous cell carcinoma (131). The molecular mechanisms underlying radiosensitization of cancer cells following EGFR inhibition is not completely clear, as it varies with the tissue of origin. Multiple mechanisms have been explored, for example, it has been shown that the radiosensitization by lapatinib is primarily mediated through inhibition of RAF/MEK/ERK pathway and combinatorial approach that also includes inhibitors of this pathway could provide superior strategy for radiosensitization in HER2-positive breast cancers (132). Other mechanisms included inhibition of EGFR nuclear transport to prevent interaction with DNAPK and inhibition of the PI3K-AKT pathway (133,134). However, not all combinatorial approaches lead to successes. Cetuximab is often combined with radiotherapy in advanced squamous cell carcinoma of the head and neck (SCCHN), but the inhibition of Src kinase by Dasatinib did not improve the efficacy of Cetuximab combined with radiotherapy (135).

BCR-ABL oncogenic fusion tyrosine kinase, mutated in 100% of CMLs and a subset of ALLs was also found to have a differential effect on the repair of DNA damage caused by various genotoxic agents. It increased the efficiency of repair in response to DNA strand breaks in BaF3 lymphoid cells, e.g. idarubicin induced DSBs were efficiently repaired in BCR-ABL-positive cells suggesting involvement of this mutant kinase in their resistance to idarubicin (136). However, BCR-ABL expressing CMLs develop resistance to the selective inhibitors such as imatinib and may rely on the alt-NHEJ pathway for repair. Consistently targeting Parp1 a member of alt-NHEJ with selective inhibitors sensitized TKI resistant BCR-ABL CMLs (137). Additionally BCR-ABL, differentially affected nucleotide excision repair in response to UV-C induced DNA damage based on the source of origin of the cell type, i.e. lymphoid vs myeloid (138). While the p210-BCR-ABL suppressed nucleotide excision repair (NER) in the BaF3 lymphoid background and correspondingly increased the mutation rates following UV-C induced damage, it conferred resistance to UV radiation in myeloid cells and primary bone marrow cells by increasing the NER activity. The UV resistance could be overcome by treatment with the selective TKI inhibitor, ST1571 (137). These observations suggest that although the oncogenic kinases are the main drivers of malignancy, their outcomes on DNA repair is influenced by additional factors.

TKIs have also been employed based on their ability to differentially suppress either the homologous recombination or the non-homologous pathway of endjoining in specific cell types and thus shown to have a therapeutic benefit. Some cell lines that mimicked the invasive bladder tumors were observed to be defective in non-homologous end-joining (NHEJ). Mechanistically, imatinib, a BCR-ABL inhibitor has an apparent inhibitory effect on the function of the HR protein, RAD51 (139). These cell lines were treated with a combination of radiotherapy and with imatinib based on the premise that inhibiting both HR and NHEJ is likely to induce toxicity. In another study, sorafenib, a potent inhibitor of RAF kinase and VEGF receptor, significantly enhanced the antiproliferative effects of chemoradiation treatment by downregulating DNA repair proteins (ERCC-1 and XRCC-1) in a dose-dependent manner (140).

Similar to many oncogenic TKs, ACK1 activation is linked to poor prognosis in a number of cancers (141) (Table 2). ACK1 tyrosine kinase not only interacts with AR in prostate cancer (Table 1) (72), but has also been shown to interact with estrogen receptor (ER) in breast cancer cells (142). Significantly, ACK1 phosphorylates the ER co-activator, KDM3A, a H3K9 demethylase, at an evolutionary conserved tyrosine 1114 site in the presence of tamoxifen, an ER antagonist (Table 1). Not surprisingly, ACK1 activation resulted in a significant decrease in the deposition of dimethyl H3K9 epigenetic marks, while its inhibition restored the repressive marks and caused transcriptional suppression of the ER-regulated genes such as the mammary tumor oncogene, *HOXA1*. Although AR is known to interact with KDM3A (143,144), whether ACK1/AR signaling utilizes KDM3A to erase the repressive H3K9 methylation marks to activate ATM expression to promote radioresistance of castration resistant prostate cancer is unknown.

Glioblastomas (GBMs) are incurable malignancies and highly resistant to radio- and chemotherapy. WEE1 and JAK2 tyrosine kinases are either upregulated or highly active in GBMs and correlated with poor prognosis (94,145). Radioresistance of GBMs is subjugated by treatment with the WEE1 inhibitor, AZD1775 (146), or the JAK2 inhibitor, AZD1480. AZD1775 is currently in clinical trials primarily due to its ability to avert the G2/M checkpoint arrest by abrogating cdc2Y15 phosphorylation to induce mitotic catastrophe in irradiated cancer cells with defective p53 (147) (Tables 1 and 2). Since these kinases have non-overlapping roles in conferring radioresistance, it remains to be seen whether combination of these two inhibitors can lead to better therapeutic outcomes.

## CONCLUSION

All the above examples, unique as they may be, illustrate the critical contribution of tyrosine kinase family of proteins in promoting DNA repair, cell cycle progression, apoptosis and maintenance of epigenetic states-processes that are fundamental to cellular homeostasis. Recent studies unraveling the evolutionary dynamics of cancer metastasis using androgen-deprived prostate cancer as a model however reinforce the requirement for a deeper understanding of the mechanics of proteins and regulators critical for the maintenance of genome stability. Genes frequently perturbed in the metastatic sub clones were those associated with the DNA repair genes, including mutations in the hypermutator mismatch repair gene, MSH2, the DNA polymerase polE and DNA repair gene *polD1*, the homologous recombination repair genes RAD52, BRCA2 and ATM, as well as missense mutations in the tumor suppressor p53 (148). AR amplification was also frequently observed in metastatic prostate cancer. AR promotes radioresistance of prostate cancers using androgen dependent or androgen independent but TK dependent mechanisms (56,149). As summarized above, many of these gene products are modified by oncogenic tyrosine kinases. Future studies exploring the cross talk of tyrosine kinases and DDR pathways during the evolutionary progression of aggressive cancers to therapy resistance and metastasis should yield a better understanding of the precise role of founder mutations and secondary mutations. Further, these studies will provide suitable rationale for designing novel targeted therapies.
